# A mechanistic evaluation of human beta defensin 2 mediated protection of human skin barrier in vitro

**DOI:** 10.1038/s41598-023-29558-0

**Published:** 2023-02-08

**Authors:** Jennifer R. Shelley, Brian J. McHugh, Jimi Wills, Julia R. Dorin, Richard Weller, David J. Clarke, Donald J. Davidson

**Affiliations:** 1 University of Edinburgh Centre for Inflammation Research, Queen’s Medical Research Institute, BioQuarter, University of Edinburgh, 47 Little France Crescent, Edinburgh, EH16 4TJ Scotland, UK; 2 Cancer Research UK Edinburgh Centre, Institute of Genetics and Cancer, The University of Edinburgh, Western General Hospital Campus, Crewe Road, Edinburgh, EH4 2XU Scotland, UK; 3 The EastChem School of Chemistry, University of Edinburgh, Joseph Black Building, Brewster Road, Edinburgh, EH9 3FJ UK; 4 Present Address: The Commonwealth Building, The Hammersmith Hospital, Du Cane Road, London, W12 0NN UK

**Keywords:** Immunochemistry, Peptides, Proteins, Inflammatory diseases, Antimicrobial responses, Immunological disorders, Inflammation, Inflammatory diseases, Skin diseases

## Abstract

The human skin barrier, a biological imperative, is impaired in inflammatory skin diseases such as atopic dermatitis (AD). *Staphylococcus aureus* is associated with AD lesions and contributes to pathological inflammation and further barrier impairment. *S. aureus* secretes extracellular proteases, such as V8 (or ‘SspA’), which cleave extracellular proteins to reduce skin barrier. Previous studies demonstrated that the host defence peptide human beta-defensin 2 (HBD2) prevented V8-mediated damage. Here, the mechanism of HBD2-mediated barrier protection in vitro is examined. Application of exogenous HBD2 provided protection against V8, irrespective of timeline of application or native peptide folding, raising the prospect of simple peptide analogues as therapeutics. HBD2 treatment, in context of V8-mediated damage, modulated the proteomic/secretomic profiles of HaCaT cells, altering levels of specific extracellular matrix proteins, potentially recovering V8 damage. However, HBD2 alone did not substantially modulate cellular proteomic/secretomics profiles in the absence of damage, suggesting possible therapeutic targeting of lesion damage sites only. HBD2 did not show any direct protease inhibition or induce expression of known antiproteases, did not alter keratinocyte migration or proliferation, or form protective nanonet structures. These data validate the barrier-protective properties of HBD2 in vitro and establish key protein datasets for further targeted mechanistic analyses.

## Introduction

Atopic dermatitis (AD) is a common and distressing disease, with significant morbidity characterised by chronic, inflamed lesions, typically on the face, scalp and limbs, affecting up to 34% of 12-year olds and 6% of 26-year olds in Northern Europe^1^. The early onset and chronic relapsing nature of AD necessitates safe, long-term use therapeutics. These therapeutics are required to rebuild skin barrier structure and prevent recurrence of the disease, while simultaneously addressing the wider-reaching implications of AD, such as alteration to the neuroendocrine function of the skin^2,3^. Topical corticosteroids, the principal treatment, can have significant detrimental effects and individuals becoming refractory to topical application can require systemic treatment^4^. The development of new therapies, based on knowledge of disease pathogenesis, is required.

A key hallmark of AD is epidermal barrier disruption. Clinical trial evidence shows that maintaining skin barrier integrity is critical in prevention of AD^5^, and highlights the potential for novel interventions that can restore barrier integrity. Barrier function is vital for the maintenance of skin health^6^ and impairment can be associated with reductions in extracellular matrix (ECM) components, such as Tight Junction (TJ) proteins and filaggrin, as well as increased pH and dysbiosis^7^. Dysbiosis associated with AD lesional skin is characterised by prominent *Staphylococcus aureus* presence^8^. This leads to cutaneous *S. aureus* infections, which represent a recurrent complication for AD patients and worsening of AD pathology, including alteration of the skin neuroendocrine output^9–11^.

*S. aureus* contributes to further skin barrier integrity breakdown through production of extracellular proteases, such as the serine protease V8 (also known as SspA)^12^. V8 significantly contributes to the pathogenicity of *S. aureus* and is known to break down skin structural proteins, such as TJs, which allows *S. aureus* to penetrate deeper into the skin and access areas with higher nutrient and moisture availability^13^. Within the deeper layers of the skin, *S. aureus* may then proliferate, leading to increased production of V8, initiating a cycle of skin structural breakdown and *S. aureus* invasion^14^.

We have previously demonstrated that application of human beta-defensin (HBD)2, an antimicrobial host defence peptide (HDP), to keratinocytes, before V8 application, provided protection against protease-mediated damage^15^. However, the mechanism of action of this peptide remained undetermined. HBD2 (gene name *DEFB4*) is a member of the positively charged HBD family^16^. Traditionally thought to have simple bactericidal activity, HBDs have, over recent years, been established as having a wide range of signalling and immunomodulatory functions^17^. HBD2 is an inducible peptide in a wide range of tissues (including the skin), with expression upregulated in response to infection, injury and IL-1β^18^ and dampened by Th2-type cytokines, such as IL-4 and IL-13^19^.

We proposed that elucidating the mechanism by which HBD2 protects keratinocyte barrier function against bacterial protease, could inform development of novel therapeutics for AD. Hypothetically, breaking the cycle of V8-mediated skin barrier damage would reduce *S. aureus* access to the deeper layers of the skin and reduce the infection burden which is common to AD patients. The direct selective antimicrobial properties of HBD2 might also contribute to reestablishment of a healthy skin microbiota^20^. As HBD2 is inducible in human skin and has been shown to prevent V8 activity^15^, it was considered a good target for exogenous application, whether in its native form or a modified version. Thus, the mechanism by which HBD2 prevented V8-mediated barrier damage was investigated, analysing both the possibility of direct protease inhibition and the impact of HBD2 on keratinocyte function in the presence and absence of V8 protease.

## Results

### HBD2 induces protection against V8-mediated barrier integrity damage

**Figure 1:**
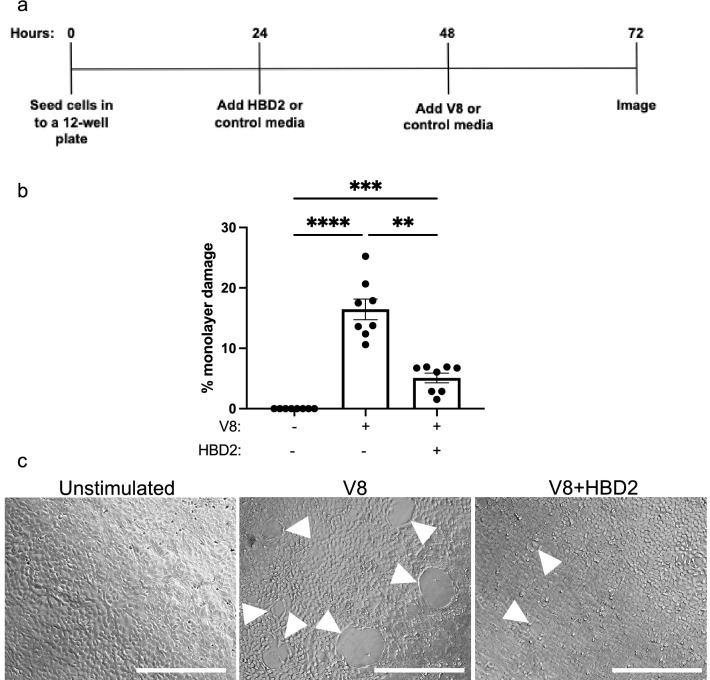
HBD2 protects against V8-mediated barrier integrity damage. (**a**) Timeline of damage assay, with treatment conditions indicated. (**b**,**c**) HaCaT cells were treated with 0.5 µg/ml HBD2 or vehicle control for 24 h. Recombinant V8 (5 µg/ml) or PBS (vehicle control) was then added, and cells were incubated for a further 24 h before 5 random fields of view were imaged per condition by phase contrast microscopy. (**b**) Quantification of V8-mediated damage represented as % monolayer damage. Data shown as mean ± SEM for n = 8. Statistical analysis by paired one-way ANOVA with Repeated Measures (RM) multiple comparisons post-test. **p < 0.01 ***p < 0.005 ****p < 0.001. (**c**) Representative images of damage at 72 h (from n = 8), with barrier integrity damage “holes” indicated with white arrow heads. Scale bar represents 200 µm.

To confirm our previous data, showing protection against V8-mediated barrier integrity damage could be achieved with exogenously applied HBD2^15^, 0.5 µg/ml of synthetic HBD2 peptide (or vehicle control media) was applied to HaCaT monolayers, before V8 application, in an established monolayer damage assay (Fig. 1a). This concentration of HBD2 was shown to provide significant protection against V8-mediated damage (Fig. 1b,c) with the large “holes”, generated by V8, minimised by prior HBD2 treatment. These “holes” have previously been shown to be unrelated to cell death, but instead likely due to protease-mediated damage of cell–cell connections. This type of damage was also shown to be explicitly reliant on the V8 protease, as *S. aureus* with genetic knock out of this protease was unable to replicate equivalent damage^15^. HBD2-mediated protection was found to be effective irrespective of whether efforts were made to wash away HBD2 before V8 treatment, if cells were incubated with HBD2 before exposure to V8, or if HBD2 and V8 were added concomitantly (Supp fig. S1). These data suggested that HBD2 may interact directly with V8 to mediate a protective antiprotease effect or could rapidly induce a protective phenotype in keratinocytes.

To examine whether native peptide structure was required for HBD2-mediated protection, which may be more suggestive of a direct antiprotease effect, additional studies utilised modified forms of synthetic HBD2, including linearised HBD2 (in which cysteines had been replaced with serines to prevent classical defensin beta-sheet stabilising disulfide bond formation) and scrambled HBD2 (a peptide with a scrambled HBD2 amino acid sequence). Significant protective properties were retained against V8 for the linearised peptide (Supp fig. S1), whereas the effect of the scrambled HBD2 was highly variable and not significantly protective. Both of these HBD2 variants, linearised and scrambled, as well as the synthetic HBD2 were characterised by mass spectrometry to confirm their molecular mass was consistent with the predicted amino acid sequence and assess their stability (Supp Table S1, Supp fig. S2).

### HBD2 does not act as an alternative substrate for V8, or as a direct protease inhibitor

**Figure 2:**
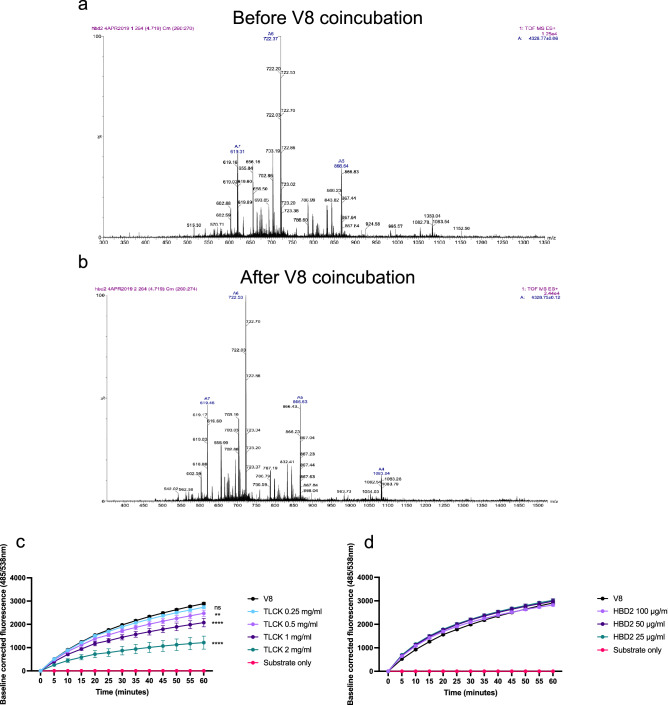
HBD2 does not act as an alternative substrate for V8. (**a**,**b**) Accurate mass measurements of synthetic HBD2 (100 µg/ml) before (**a**) and after (**b**) 1-h coincubation with V8 (12.5 µg/ml). Peptides were electrosprayed at a concentration of 50 µg/ml from a solution of 50:49:1 water/methanol/acetic acid (v/v/v). (**c**,**d**) FITC-labelled casein substrate was used to measure proteolytic activity of V8 in the presence or absence of either (**c**) 0.25–2 mg/ml TLCK or (**d**) 25–100 μg/ml HBD2. Fluorescence was measured at 485/538 nm every 5 min for 1 h and corrected by subtraction of the background fluorescence from the 0-min timepoint. HBD2 data were also normalized for low level HBD2 autofluorescence. 12.5 μg/ml V8 and 5 μg/ml substrate were used throughout. Data represents mean ± SEM for n = 5. Statistical analysis using Mann–Whitney test of final timepoints. ***p < 0.0001.

To investigate whether HBD2 was protecting monolayers by acting as an alternative substrate for V8, LC–MS was conducted on HBD2 before and after incubation with V8. The resulting spectra showed no change in the peptide following coincubation with V8 protease (Fig. 2a,b), indicating that HBD2 was not acting as an alternative substrate.

To investigate the alternative possibility that HBD2 was acting as a protease inhibitor, a cell-free, functional protease assay was established, using Pierce Fluorescent Protease Assay Kit. The system was validated with a known protease inhibitor Tosyl-L-lysyl-chloromethane hydrochloride (TLCK). TLCK was added, in a concentration curve, with 12.5 µg/ml of V8 to cleave the substrate (Fig. 2c). There was a clear progressive reduction in V8 activity across the increasing concentration of TLCK, over 1-h incubation. A HBD2 concentration curve was then applied, substituting TLCK. This showed no direct inhibition of V8 proteolysis at any concentration of HBD2 (Fig. 2d).

### HBD2 does not modulate scratch wound repair or ultrastructural appearance of HaCaT monolayers

In the absence of any direct HBD2-mediated antiprotease effect, the potential for modulation of wound healing, observed with other HDP^21–24^ was then considered. HBD2 treatment was evaluated in a HaCaT scratch healing assay (Supp fig. S3). Treatment with HBD2 before or after wounding had no effect on scratch wound healing rate. This indicated that HBD2-mediated protection was not the result of altered keratinocyte repair dynamics.

Given that HBD2 had no direct effect on V8 proteolytic function but was protective even after cells had been washed to remove exogenously applied HBD2, before V8 treatment, the interaction between cells and peptide was examined using a fluorescently labelled HBD2 (TAMRA-HBD2). First, to validate use of TAMRA-HBD2, retention of peptide-mediated barrier-protective function against V8 was confirmed for TAMRA-HBD2 (Supp fig S4). Microscopic studies were then conducted to examine peptide localisation, demonstrating that washing of the monolayer did not entirely remove the TAMRA-HBD2 (although it was reduced (Supp fig S4)). Diffuse peptide was visible across the surface of the monolayer in TAMRA-HBD2-treated cells, in addition to bright foci. V8 addition had no substantive impact on quantified fluorescence (Supp fig S4). These data suggest that peptide binds and/or internalises into cells, potentially creating a protective barrier on the apical cell surface and/or modifying cell functions.

**Figure 3:**
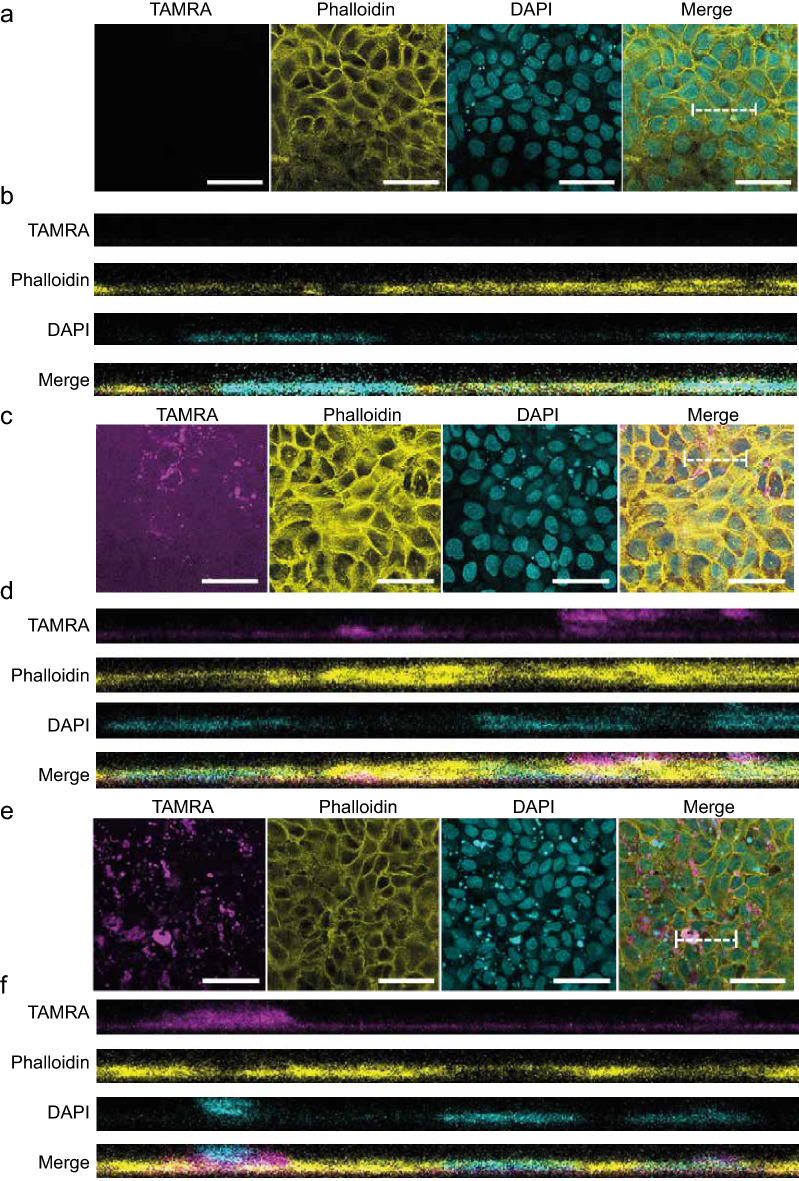
Fluorescent HBD2 localization. HaCaT cells were seeded into ibidi 8-well chamberwell slides. After confluency was reached, cells were treated with vehicle control (**a**,**b**) or 0.5 μg/ml TAMRA-labelled synthetic HBD2 (**c**–**f**). Images were collected 48 h after addition of peptide. Cells were fixed in 4% PFA and stained with Alex488 phalloidin (shown as yellow) and Hoechst (shown as cyan) with TAMRA HBD2 (shown as magenta). Images taken with Leica SP8 confocal microscope at 20 × magnification, with z-stacks taken at 0.7 μm intervals. 5 images taken per condition per time point. Representative of n = 2 biological repeats. (**a**,**c**,**e**) Representative images of one quarter confocal fields of view. Scale bar represents 50 μm. Dotted white lines indicate areas represented in 3D renderings. (**b**,**d**,**f**) Representative images of 3D rendering, generated with LASX software.

To further characterise localisation of HBD2 within the HaCaT monolayer, confocal microscopy with subsequent 3D rendering was employed (Fig. 3). Cells were seeded in Ibidi chamber slides and stained for phalloidin (yellow) and cell nuclei (DAPI; blue) in addition to visualisation of TAMRA-HBD2 (magenta). Z-stack images were taken of fields of view at 0.7 µm intervals and projected in 3D rendered images using LASX microscopy software. Representative images of unstimulated monolayers (Fig. 3a,b) show the flattened nature of HaCaT cells, with somewhat uneven distribution of nuclei within the monolayer and some condensed and fragmented nuclear material (compatible with the background level of apoptosis). When TAMRA-HBD2 was added, fluorescent focal points of intense accumulation of peptide, similar to those in the EVOS images (Supp fig S4) were clearly observed, with seemingly random distribution (Fig. 3c–f). There was also a variable level of diffuse fluorescence, not visible in the unstimulated cells, compatible with a distribution of peptide across the monolayer, with an uneven distribution apically, intracellularly and at a low level basally (Fig. 3d). In some areas TAMRA-HBD2 was intensely focused around certain nuclei (Fig. 3f). None of these observations were uniform throughout the monolayer, therefore, two sets of representative images are used illustratively; firstly, in an area of broad TAMRA fluorescence (Fig. 3c,d), in which both apical and basal distribution is observed, and secondly in an area with more focal points or ‘speckling’ (Fig. 3e,f). In the latter, the cells which had intense TAMRA fluorescence around the nuclei, tended to have pyknotic condensed (DAPI-intense) nuclei, potentially with more superficial cells being shed from the monolayer, indicating possible apoptosis in these cells. In both sets of images, a small layer of TAMRA fluorescence is visible, seemingly underneath the cell monolayer (Fig. 3d,f). Again, this appears quite diffuse and at a low level of fluorescence but is more consistent across both representative image sets. Overall, despite absence of a consistent pattern of distribution (other than diffuse low level monolayer coverage), importantly, these data show that HBD2 was not specifically localised to the TJ regions that might be targeted by V8.

**Figure 4:**
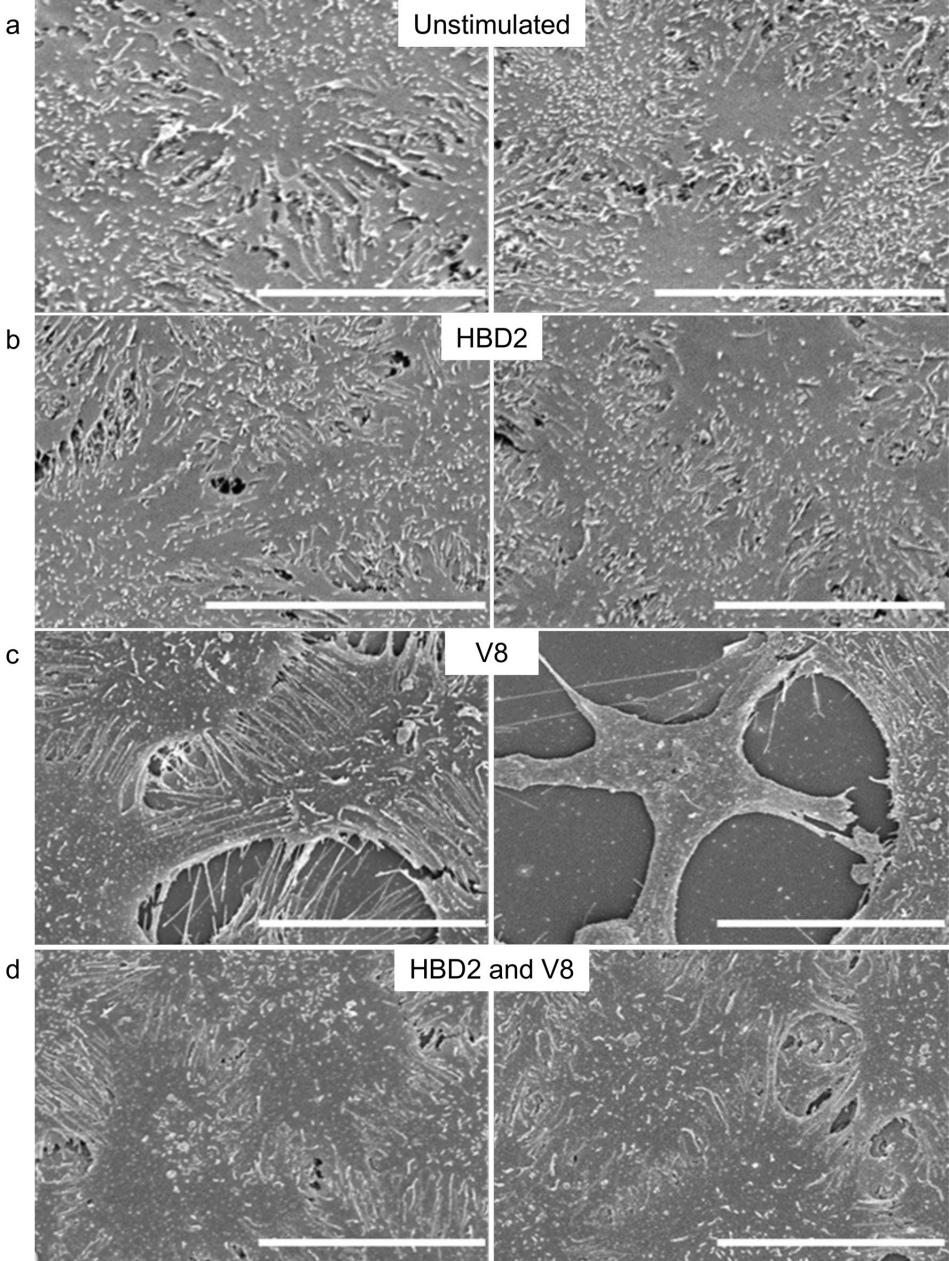
Scanning electron microscopic imaging of HaCaT cells exposed to HBD2 and/or V8. HaCaT cells were seeded into 35 mm corning coated TC dishes. Once confluency was reached, cells were treated with vehicle control (**a**,**c**) or 0.5 μg/ml synthetic HBD2 (**b**,**d**). After 24 h, cells were washed and then exposed to vehicle control (**a**,**b**) or 5 μg/ml recombinant V8 (**c**,**d**) for an additional 24 h, before cells were fixed with PFA and glutaraldehyde buffer overnight at 4 °C. Cells were then washed; glutaraldehyde buffer was added for a further overnight incubation at 4 °C and prepared for imaging. Samples imaged with the Hitachi S-4700 scanning electron microscope. Scale bars represent 20 μm. Images representative of 5 images taken per condition.

The diffuse appearance of the TAMRA-HBD2 led to consideration of a nanonet-like function, as previously described for Human Defensin 6, and for a reduced form of HBD1^25,26^. The possibility that HBD2 could form similar ultrastructures on the HaCaT monolayer apical cell surface, with protective function, possibly preventing V8 access to substrates was therefore examined by SEM (Fig. 4a–d). Treatment with HBD2 alone had no visible effect on cells (Fig. 4b), with no nanonet structures observed. Exposure to V8 alone showed a stark demonstration of the damage caused by this protease, with cells appearing to be pulled apart from one another, creating holes/wounds (evident here at a much more microscopic damage level; Fig. 4c). Protection with HBD2 minimised evidence of damage, however there was no evidence of HBD2 nanonet or other ultrastructure formation under any conditions (Fig. 4).

### Investigating the impact of HBD2 on the protein profile of HaCaT cells, in the absence of V8 proteolytic challenge

Having concluded that HBD2 did not act through direct antiprotease activity (Fig. 2), modulation of wound healing (Supp fig. S3), or formation of protective ultrastructures on the monolayer (Fig. 3 + 4), focus turned to understanding the potential impact of HBD2 on HaCaT keratinocyte function. To assess this in an unbiased manner, a global secretomics and proteomics approach was taken.

**Figure 5:**
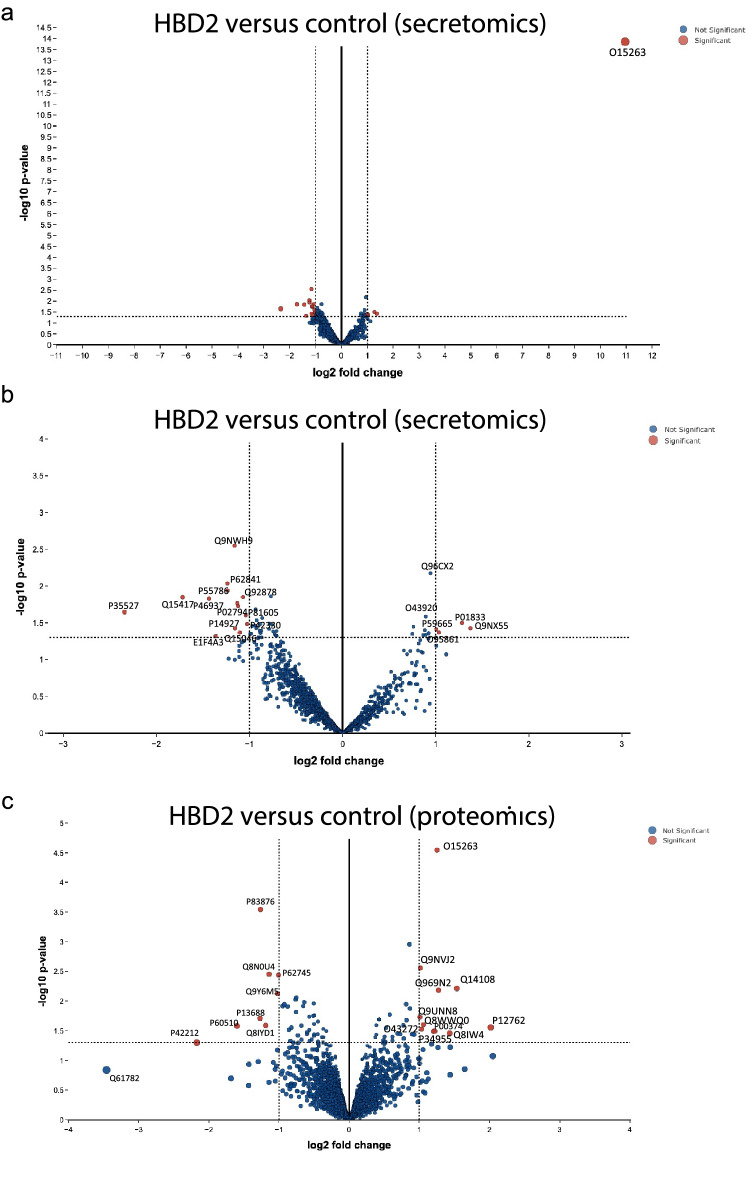
Secretomic and proteomic analyses of HBD2-stimulated HaCaT cells. HaCaT cell monolayers were treated with 0.5 μg/ml HBD2, or vehicle control, in serum free media for 48 h before supernatants (**a**,**b**) and cells (**c**) were collected and centrifuged at 3000 rpm for 5 min. Media (**a**,**b**) and dry cell pellet (**c**) were stored at -80 °C prior to analysis. Data representative of n = 5. Log fold changes and p values generated using LIMMA pathway^37^. (**a**,**b**) Volcano plots generated from secretomics analysis of HBD2-stimulated HaCaT cells compared to control vehicle-treated cells. Red points indicate significant points, blue indicate nonsignificant points. (**a**) Data represented as a whole. (**b**) Data represented without O15263 data point, zoomed in to central portion of graph. (**c**) Volcano plot generated from proteomics analysis of HBD2-stimulated HaCaT cells compared to control vehicle-treated cells. Red points indicate significant points, blue indicate nonsignificant points.

First, it was important to understand the impact of HBD2 alone on the HaCaT monolayer, independent of any V8 activity. Therefore, secretomics (Fig. 5a,b) and proteomics (Fig. 5c) analyses were conducted on HaCaT supernatant and cell pellets, respectively, following 48 h of HBD2 stimulation (replicating the exposure period used in earlier experiments; Fig. 5). LIMMA pathway analysis of the secretomics dataset confirmed detection of exogenously applied HBD2 (O15623, DEFB4A) at a high level in supernatants (Fig. 5a), providing a positive control system validation. For improved visualisation of other proteins present, a volcano plot without DEFB4A was also generated (Fig. 5b). Protein levels that differed between conditions by > 1.0 log2 fold change of and with a *p* < 0.05 were considered significantly different and of interest. Tables of significantly differentially detected proteins, following HBD2 application, were created for both the secretomics (Table 1) and proteomics (Table 2) analyses.

**Table 1:** Secretomic analysis of HBD2-stimulated HaCaT cells.

Protein code	Gene	Log2 fold change	p.mod	Protein name
O15263	DEFB4A	10.96	0.00	Beta-defensin 4A
Q9NX55	HYPK	1.37	0.04	Huntingtin-interacting protein K
P01833	PIGR	1.28	0.03	Polymeric immunoglobulin receptor
O95861	BPNT1	1.03	0.04	3'(2'),5'-bisphosphate nucleotidase 1
P59666	DEFA1/3	1.01	0.04	Neutrophil defensin 3
Q96CX2	KCTD12	0.94	0.01	BTB/POZ domain-containing protein KCTD12
O43920	NDUFS5	0.89	0.03	NADH dehydrogenase [ubiquinone] iron-sulfur protein 5
P42330	AKR1C3	− 1.04	0.02	Aldo–keto reductase family 1 member C3
Q92878	RAD50	− 1.07	0.01	DNA repair protein RAD50
Q15046	KARS	− 1.10	0.04	Lysine–tRNA ligase
P02794	FTH1	− 1.12	0.02	Ferritin heavy chain
P81605	DCD	− 1.13	0.02	Dermcidin
P14927	UQCRB	− 1.16	0.04	Cytochrome b-c1 complex subunit 7
Q9NWH9	SLTM	− 1.16	0.00	SAFB-like transcription modulator
P55786	NPEPPS	− 1.24	0.01	Puromycin-sensitive aminopeptidase
P62841	RPS15	− 1.24	0.01	40S ribosomal protein S15
P38919	E1F4A3	− 1.36	0.05	Eukaryotic initiation factor 4A-III
P46937	YAP1	− 1.43	0.01	Transcriptional coactivator YAP1
Q15417	CNN3	− 1.72	0.01	Calponin-3
P35527	KRT9	− 2.34	0.02	Keratin, type I cytoskeletal 9

HaCaT cell monolayers were treated with 0.5 μg/ml HBD2, or vehicle control, in serum free media for 48 h before supernatants were collected, centrifuged at 3000 rpm for 5 min, and stored at -80 °C prior to analysis; n = 5 per condition. Log fold changes and p values were generated using LIMMA pathway^43^. Table shows proteins with the greatest differences in detection levels between control and HBD2 treated conditions, listed by fold change, largest to smallest, with Uniprot protein code, gene code, fold change, p.mod value, protein name and brief description of function. Protein descriptions made using UniProt database information^46^.

**Table 2:** Proteomic analysis of HBD2-stimulated HaCaT cells.

Protein code	Gene	Log2 fold change	p.mod	Protein name
P12763	Bovine AHSG	2.02	0.03	Bovine Alpha-2-HS-glycoprotein
Q14108	SCARB2	1.53	0.01	Lysosome membrane protein 2
Q8IWA4	MFN1	1.43	0.03	Mitofusin-1
Q969N2	PIGT	1.27	0.01	GPI transamidase component PIG-T
O15263	DEFB4A	1.25	0.00	Beta-defensin 4A
P00374	DHFR	1.22	0.03	Dihydrofolate reductase
P34955	Bovine SERPINA1	1.20	0.03	Bovine Alpha-1-antiproteinase
Q8WWQ0	PHIP	1.06	0.03	PH-interacting protein
O43272	PRODH	1.03	0.03	Proline dehydrogenase 1, mitochondrial
Q9NVJ2	ARL8B	1.01	0.00	ADP-ribosylation factor-like protein 8B
Q9UNN8	PROCR	1.01	0.02	Endothelial protein C receptor
P62745	RHOB	− 1.01	0.00	Rho-related GTP-binding protein RhoB
Q9Y6M5	SLC30A1	− 1.02	0.01	Zinc transporter 1
Q8N0U4	FAM185A	− 1.14	0.00	Protein FAM185A
Q8IYD1	GSPT2	− 1.19	0.03	Eukaryotic peptide chain release factor GTP-binding subunit ERF3B
P83876	TXNL4A	− 1.27	0.00	Thioredoxin-like protein 4A
P13688	CEACAM1	− 1.27	0.02	Carcinoembryonic antigen-related cell adhesion molecule 1
P60510	PPP4C	− 1.60	0.03	Serine/threonine-protein phosphatase 4 catalytic subunit
P42212	GFP	− 2.17	0.05	Jellyfish Green fluorescent protein
Q61782	Mouse Q61782	− 3.46	0.14	Mouse Type I epidermal keratin mRNA

HaCaT cell monolayers were treated with 0.5 μg/ml HBD2, or vehicle control, in serum free media for 48 h before the cells were washed rigorously with PBS and scraped from the well surface. Cell pellets were washed an additional three times with PBS in suspension. Dry cell pellets were stored at -80 °C prior to analysis; n = 5 per condition. Log fold changes and P values generated using LIMMA pathway^43^. Table shows proteins with the greatest differences in detection levels between control and HBD2 treated conditions, largest to smallest with Uniprot protein code, gene code, fold change, p.mod value, protein name and brief description of function. Protein descriptions made using UniProt database information^46^.

In the secretomics analysis, only a small number of proteins (seven) were detected at significantly greater levels in the HBD-treated samples, and it should be noted that these were very close to the threshold of significance (Table 1). Importantly, there were no clear differences in proteins associated with keratinocyte proliferation or migration, compatible with the observations on scratch wound healing. There were more proteins for which detected levels were lower in the supernatants of HBD2-treated cells. Several of these were associated with basic cell metabolism. Also of note were the significantly lower levels of Dermcidin (P81605, DCD).

Proteomics analysis also revealed a small number of proteins with altered levels (eleven increased, nine decreased) following HBD2 application. Of those with a higher level detected in response to HBD2, several are involved in lysosome activity and basic cell function, and PH-interacting protein (Q8WWQ0, PHIP), which is involved in proliferation (Table 2). HBD2 (O15263, DEFB4A) was again identified as one of the most differentially detected proteins, compatible with imaging data that suggested cell binding and internalisation of exogenously added peptide (Fig. 3). Of the proteins which were detected at lower levels in HBD2-treated cells (Table 2), carcinoembryonic antigen-related cell adhesion molecule 1 (P13688, CEACAM1) was of interest, due to its involvement in cell adhesion.

Overall, the impact of HBD2 on the undamaged monolayer was low, with no substantial or revealing impact on HaCaT cells in the absence of a V8 proteolytic challenge, concurring with the earlier data (Supp fig. S3, Fig. 3).

### Investigating the impact of HBD2 on the protein profile of HaCaT cells, in the context of V8-mediated barrier integrity damage

Given that HBD2 had minimal impact on a healthy monolayer (Fig. 5, Tables 1, 2), the focus shifted to investigating whether the mechanism underpinning HBD2-mediated protection in this system was manifested only in the context of V8-mediated damage. This led to two hypotheses: that HBD2 induced production of protease inhibitors from HaCaT cells during proteolytic challenge, or that HBD2 induced production of TJ and/or ECM proteins in the context of V8-mediated barrier integrity damage. To investigate these hypotheses, secretomics and proteomics analyses were used to compare the protein profile of HaCaT keratinocytes exposed to V8 damage, with or without prior macroscopic protection mediated by HBD2 application. Prior to secretomics and proteomics analyses, it was necessary to demonstrate that the cell cultures used for these analyses demonstrated the HBD2-protective phenotype. Cells that were used for ‘omics analyses were therefore imaged and analysed before use (Supp fig. S5). These cells were shown to demonstrate the protective phenotype.

To investigate the mechanism of HBD2-mediated protection in the context of V8, the effect of V8-alone on the HaCaT keratinocytes was first determined. Although V8 cleavage of TJ and ECM proteins in cell culture has been described (Hirasawa et al., 2010), the global impact on proteins, mediated by V8, has not been characterised. Therefore, secretomics and proteomics were conducted on HaCaT cells exposed to V8 alone. Data compared to unstimulated cells are provided (Supp fig. S6, Supp table S2 + S3). This established a baseline for further comparative analyses.

**Figure 6:**
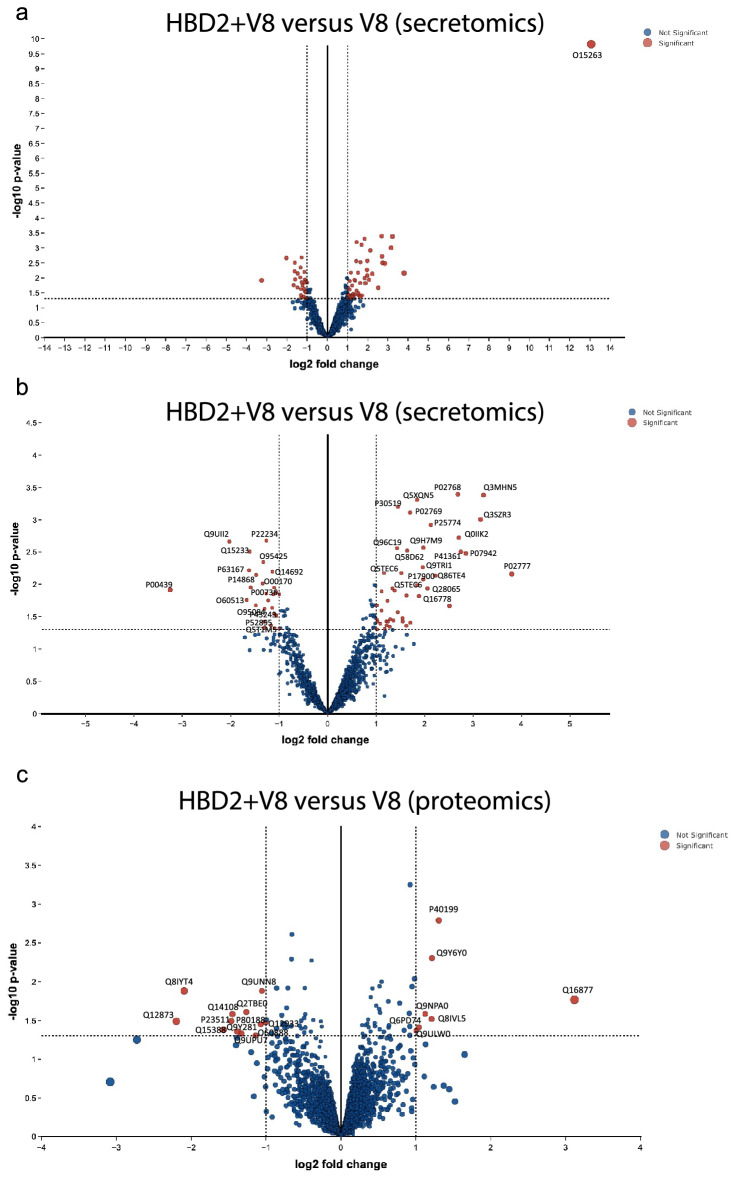
Secretomic and proteomic characterisation of HBD2-mediated protection from V8 damage in HaCaT cells. HaCaT cell monolayers were treated with 0.5 μg/ml HBD2 in serum free media for 24 h, before addition of 5 μg/ml V8, or vehicle control, for a further 24 h. Supernatants (**a**,**b**) and cells (**c**) were then collected, centrifuged at 3000 rpm for 5 min, and stored at -80 °C prior to analyses. n = 5 per condition. Log fold changes and p values generated using LIMMA pathway^43^. (**a**,**b**) Volcano plots generated from secretomics analysis from the supernatants of HaCaT cells protected with HBD2 before exposure to V8, compared to unprotected V8-damaged cells. Red points indicate significant points, blue indicate nonsignificant points. (**a**) Data represented as a whole. (**b**) Data represented without O15263 data point, zoomed in to central portion of graph. (**c**) Volcano plot generated proteomics analysis of HaCaT cells protected with HBD2 before exposure to V8, compared to unprotected V8-damage cells. Red points indicate significant points, blue indicate nonsignificant points.

Having characterised the supernatant and cellular proteins following application of V8-alone, as a damage-specific baseline dataset, the secretome and proteome profiles generated by a combined HBD2 and V8 application were analysed as a direct comparison to the V8-alone condition (Fig. 6). As in the HBD2-only dataset, this secretomics analysis highlighted HBD2 (Beta defensin 4A; O15263; DEFB4A) as the most abundant protein (Fig. 6a). As its high magnitude of change skewed the volcano plot, a second plot was constructed without DEFB4A (Fig. 6b). Of interest in this secretome dataset (Table 3) were the higher levels of Laminin subunit beta-1 (P07942; LAMB1) in the HBD2-protected samples. LAMB1 is a member of the Laminin family, which form a vital part of the ECM in skin. This could contribute to the second hypothesis for HBD2-mediated protection, indicating that HBD2 modulated levels of ECM proteins in the context of V8 damage. It is important to note here that no induction was identified of any known protease inhibitor in the context of HBD2-protected V8-treated cells.

**Table 3:** Secretomic analysis of HBD2-mediated protection from V8 damage in HaCaT cells.

Protein code	Gene	Log2 fold change	p.mod	Protein name
O15263	DEFB4A	13.06	0.00	Beta-defensin 4A
P02777	Bovine PF4	3.80	0.01	Platelet factor 4
Q3MHN5	Bovine GC	3.22	0.00	Vitamin D-binding protein
Q3SZR3	Bovine ORM1	3.16	0.00	Alpha-1-acid glycoprotein
P07942	LAMB1	2.85	0.00	Laminin subunit beta-1
P41361	Bovine SERPINC1	2.75	0.00	Antithrombin-III
Q0IIK2	Bovine TF	2.71	0.00	Serotransferrin
P02768	ALB	2.69	0.00	Albumin
Q86TE4	LUZP2	2.22	0.01	Leucine zipper protein 2
P25774	CTSS	2.13	0.00	Cathepsin S
Q28065	Bovine C4BPA	2.06	0.01	C4b-binding protein alpha chain
Q9H7M9	C10orf54	1.98	0.00	V-type immunoglobulin domain-containing suppressor of T-cell activation
P17900	GM2A	1.98	0.01	Ganglioside GM2 activator
Q9TRI1	N/A	1.96	0.01	Inter-alpha-trypsin inhibitor HC2 component homolog
Q16778	H2BC21	1.88	0.02	Histone H2B type 2-E
Q5XQN5	Bovine KRT5	1.85	0.00	Keratin, type II cytoskeletal 5
Q5TEC6	HIST2H3PS2	1.85	0.01	Histone H3
P02769	Bovine ALB	1.70	0.00	Albumin
Q58D62	Bovine FETUB	1.64	0.00	Fetuin-B
P30519	HMOX2	1.45	0.00	Heme oxygenase 2
Q96C19	EFHD2	1.44	0.00	EF-hand domain-containing protein D2
P00978	Bovine AMBP	1.16	0.01	Protein AMBP
Q9UII2	ATPIF1	− 2.03	0.00	ATPase inhibitor, mitochondrial
P00439	PAH	− 3.25	0.01	Phenylalanine-4-hydroxylase
P63167	DYNLL1	− 1.63	0.01	Dynein light chain 1, cytoplasmic
O60513	B4GALT4	− 1.67	0.02	Beta-1,4-galactosyltransferase 4
Q15233	NONO	− 1.61	0.00	Non-POU domain-containing octamer-binding protein
P14868	DARS	− 1.48	0.01	Aspartate–tRNA ligase, cytoplasmic
O95084	PRSS23	− 1.48	0.02	Serine protease 23
O95425	SVIL	− 1.33	0.00	Supervillin
O00170	AIP	− 1.34	0.01	AH receptor-interacting protein
P22234	PAICS	− 1.27	0.00	Multifunctional protein ADE2
P00736	C1R	− 1.27	0.01	Complement C1r subcomponent
Q5T1M5	FKBP15	− 1.30	0.05	FK506-binding protein 15
P43243	MATR3	− 1.31	0.02	Matrin-3
P52895	AKR1C2	− 1.31	0.04	Aldo–keto reductase family 1 member C2
Q14692	BMS1	− 1.14	0.01	Ribosome biogenesis protein BMS1 homolog

HaCaT cell monolayers were treated with 0.5 μg/ml HBD2 in serum free media for 24 h, before addition of 5 μg/ml V8, or vehicle control, for a further 24 h. Supernatants were then collected, centrifuged at 3000 rpm for 5 min and stored at -80 °C prior to analysis. n = 5 per condition. Log fold changes and p values generated using LIMMA pathway^43^. Table shows proteins with the greatest differences in detection levels between HBD2-protected and unprotected cells after exposure to V8, largest to smallest, with Uniprot protein code, gene code, fold change, p.mod value, protein name and brief description of function. Protein descriptions made using UniProt database information^46^.

In the proteomics analysis (Fig. 6c, Table 4) Carcinoembryonic antigen-related cell adhesion molecule 6 (P40199; CEACAM6) was identified at higher levels in V8-treated cells protected with HBD2, compared to V8 exposure alone, as well as proteins involved in cell metabolism. The biological significance in the changes of the majority of these metabolism-associated proteins remains to be determined. LEPREL1, however, was noteworthy given it’s involvement in collagen production. CEACAM6 was also considered interesting, as it is known to contribute to cell adhesion. These data further support the hypothesis that HBD2 modulates ECM protein levels in the context of V8-mediated damage.

**Table 4:** Proteomic analysis of HBD2-mediated protection from V8 damage in HaCaT cells.

Protein code	Gene	Log2 fold change	p.mod	Protein name
Q16877	PFKFB4	3.12	0.02	6-phosphofructo-2-kinase/fructose-2,6-bisphosphatase 4
P40199	CEACAM6	1.31	0.00	Carcinoembryonic antigen-related cell adhesion molecule 6
Q9Y6Y0	IVNS1ABP	1.21	0.00	Influenza virus NS1A-binding protein
Q8IVL5	LEPREL1	1.21	0.03	Prolyl 3-hydroxylase 2
Q9NPA0	EMC7	1.12	0.03	ER membrane protein complex subunit 7
Q6PD74	AAGAB	1.04	0.04	Alpha- and gamma-adaptin-binding protein p34
Q9ULW0	TPX2	1.00	0.04	Targeting protein for Xklp2
Q12873	CHD3	-2.20	0.03	Chromodomain-helicase-DNA-binding protein 3
Q8IYT4	KATNAL2	-2.09	0.01	Katanin p60 ATPase-containing subunit A-like 2
Q15388	TOMM20	-1.57	0.04	Mitochondrial import receptor subunit TOM20 homolog
P23511	NFYA	-1.47	0.03	Nuclear transcription factor Y subunit alpha
Q14108	SCARB2	-1.45	0.03	Lysosome membrane protein 2
Q9Y281	CFL2	-1.38	0.04	Cofilin-2
Q9UPU7	TBC1D2B	-1.33	0.05	TBC1 domain family member 2B
Q2TBE0	CWF19L2	-1.26	0.02	CWF19-like protein 2
O60888	CUTA	-1.14	0.05	Protein CutA
P80188	LCN2	-1.07	0.04	Neutrophil gelatinase-associated lipocalin
Q9UNN8	PROCR	-1.06	0.01	Endothelial protein C receptor
Q12933	TRAF2	-1.01	0.03	TNF receptor-associated factor 2

HaCaT cell monolayers were treated with 0.5 μg/ml HBD2 in serum free media for 24 h, before addition of 5 μg/ml V8, or vehicle control, for a further 24 h. Cells were then collected and centrifuged at 3000 rpm for 5 min. Dry cell pellet stored at -80 °C prior to analysis. n = 5 per condition. Log fold changes and p values generated using LIMMA pathway^43^. Table shows proteins with the greatest differences in detection levels between HBD2-protected and unprotected cells after exposure to V8, largest to smallest with Uniprot protein code, gene code, fold change, p.mod value, protein name and brief description of function. Protein descriptions made using UniProt database information^46^.

## Discussion

The need for new, safe, long-term treatments for AD is a large-scale global requirement. Novel interventions that target barrier integrity were identified as a top-10 translational dermatology research priority by researchers, clinicians, patients and policy makers in a e-Delphi exercise^27^. AD lesions represent a breakdown in barrier function which allow for increased incidence of cutaneous infection, particularly by the bacteria *S. aureus*^9^, which produces extracellular proteases (such as V8), allowing deeper bacterial penetration and worsening of AD pathology^28^. We proposed that elucidating the mechanisms underpinning the barrier-protective properties of HBD2, against V8, could inform future therapeutic and/or preventative strategies for AD and other chronic inflammatory skin diseases.

These studies confirmed the phenotype of HBD2-mediated protection in a HaCaT cell monolayer model and demonstrated the capacity of HBD2 to provide protection, regardless of different application timelines and without the requirement for native peptide folding. Further assays demonstrated that HBD2 did not inhibit the proteolytic function of V8 in a cell-free environment and was not permanently altered by the protease. These observations led to a focus on the hypothesis that HBD2 acts directly upon the keratinocytes to modify cellular processes. HBD2 did not have any impact on keratinocyte migration or proliferation, specifically localise into the monolayer in clearly defined or instructive pattern, or form ultrastructures, visible by SEM. Although the possibility that HBD2 ultrastructures were washed off during SEM sample processing cannot be excluded, this was not considered an avenue for further investigation. Proteomics and secretomics analyses^29^ conducted on HBD2-stimulated monolayers, both independently and alongside V8 application, concluded that HBD2 did not induce production of known protease inhibitors, but did modulate levels of ECM proteins in the context of V8-mediated barrier integrity damage.

The initial concept underlying these studies was that HBD2 could be employed as a direct antiprotease to prevent or treat *S. aureus* V8-mediated skin damage, with additional direct selective microbicidal properties that might help to address skin dysbiosis in individuals with AD. However, the initial experiments clearly excluded a direct effect of the peptide on V8 proteolytic function and found no evidence of nanonets or HBD2 localisation at cellular junctions to suggest focused barriers to protease damage. These results led to redirection of the experimental approach, to evaluating HBD2-mediated effects on keratinocyte function. An indirect protective effect, via HBD2-mediated induction of alternative antiproteases was an attractive hypothesis, potentially targeting serine protease inhibitors found in the skin, such as Lympho-epithelial Kazal-type-related inhibitor (LEKTI), antileukoproteinase or secretory leukocyte protease inhibitor (SLPI) and elafin^30–32^. Each of these serine protease inhibitors are inducible in the skin^33^. However, no evidence of this was found in proteomic or secretomic studies of protected cell cultures, suggesting an alternative mechanism must be responsible for the barrier protective properties that are observed here with HaCaT cells.

The nature of the V8-mediated barrier damage suggested that modulation of TJ or ECM proteins might have the potential to compensate for, or correct, the proteolytic damage phenotype. Therefore, the impact of HBD2 on keratinocyte protein production was evaluated, taking an unbiased proteomic and secretomics screening approach. Given that these effects might not be visible when HBD2 was applied alone to a healthy monolayer (as sufficient levels of these proteins would already exist), treatment was studied both in the presence and absence of V8. It is important to note that the results of these studies must be considered in the context of the preparation process. As this relies on identification of protein fragments specifically following trypsin cleavage, it was unlikely that proteins which had been cleaved by V8 would be identifiable in the supernatant. Therefore, lower cell pellet protein levels after V8 treatment, in comparison to undamaged cells, was taken to represent proteolytic destruction, rather than higher levels of cleaved fragments in the supernatant. Prevention of this loss in the presence of HBD2 was taken to demonstrate protective modulation of these proteins, by a reactive increase in synthesis or prevention of proteolysis.

Proteomic analysis of HBD2-stimulated undamaged monolayers did not demonstrate any substantial modulation of keratinocyte protein profiles, although alteration in proteins involved in basic cell metabolism and lysosomal function were observed. This was compatible with the mechanical wound healing assays, which showed no impact of HBD2 in the absence of protease-mediated damage. That contrasts with some previous studies, which demonstrated peptide-mediated enhanced migration and/or proliferation in both fibroblasts and keratinocytes^21–23^. However, the concentrations of peptide used in our study were much lower than those used in these publications, which showed induction of proliferation and migration of keratinocytes in response to exposure to 20 µg/ml of HBD2^21,23^. The lower concentrations used here were considered more physiologically relevant for human AD skin (quantified at 2.7 μg/ml), although HBD2, overexpressed in psoriatic skin, can reach 50 µg/ml^34,35^. In addition, in our earlier study, demonstrating that IL-1β-mediated protection against V8-induced damage was secondary to HBD2 induction, IL-1β-mediated production of 300 pg/ml of HBD2 (or 0.03 µg/ml) was protective.

The identification of HBD2 at very high levels in these supernatants was a useful technical validation of the proteomics analysis and was compatible with the cellular integration of HBD2 identified by confocal imaging and likelihood of cell internalisation, as is commonly seen with other HDP^36,37^. These analyses suggest that the impact of HBD2 on the undamaged monolayer is low. Given that overexpression has been proposed to contribute to pathogenesis in psoriasis development^17^, the observation that the levels of HBD2, which are protective against V8, have minimal impact of healthy keratinocytes, is encouraging when considering the therapeutic potential of such peptides.

Having concluded that, in this model system, HBD2-mediated protection against proteolytic barrier integrity damage was not a consequence of protein modulation prior to exposure to protease, attention then turned to the damage model. Analysis of these samples demonstrated that levels of ECM proteins, specifically LAMB1 in the secretomics and CEACAM6, were higher in V8 treated cultures with macroscopically demonstrated HBD2-mediated protection from barrier integrity damage, when compared to those exposed to V8 alone. LEPREL1, which is involved in collagen production, was also higher. This suggested HBD2-mediated modulation of ECM protein levels, replacing and/or preventing the loss of these ECM proteins caused by V8 activity, and associated with prevention of formation of V8-associated wounds. This differed from the higher levels of certain proteins associated with cell adhesion, such as DDR1, tenascin and angiomotin observed as a direct response to V8 damage alone. Although the mechanisms underpinning this ECM protein ‘boosting’ of the cellular response to the protease now needed to be determined, these data sets signpost future research towards the development of possible novel interventions for AD.

An important caveat of this study is the cell type which was used throughout. Although our previous work demonstrated that the V8 damage and HBD2 protective phenotypes were also observed in human primary epidermal keratinocytes^15^, this current study was carried out exclusively with HaCaT cells. This is a monolayer cell line culture and, although very commonly used, it is not clear which strata of the epidermis it best represents. In the proteomics analysis, multiple proteins which would be considered ‘characteristic’ of different layers of the epidermis were identified, ranging from collagen (found in the Stratum Basale and Basement Membrane) to involucrin (in the Stratum Corneum and somewhat in the Stratum Granulosum). It is also recognised that the impact of HBD2 on additional dermatological cell types, including fibroblasts, surveillant immune cells and neurones could alter the mechanism of HBD2 action. Therefore, validation of the key observations from the proteomics analyses will now need to be conducted using primary cells and in 3D skin model system, in conjunction with further dissection of the underlying mechanisms involved.

In addition to illuminating targets and pathways relevant to fully understanding the protective properties of HBD2, these data also provide additional potential screening endpoints to examine peptide libraries for smaller, simpler peptide derivatives with therapeutic potential. The observation that linearised HBD2 retained protective function, demonstrated that the native conformation (stabilised by three disulphide bridges^17^) is unnecessary, simplifying synthesis on a larger scale. Similarly, linearised β-defensins have been shown to retain antimicrobial activity, with HBD3 folding variants shown to be active against *Escherichia coli* ^38^, confirmed by another study demonstrating linearised HBD3 had greater activity against gram-negative bacteria than the folded peptide^39^. This potential to modify defensins while retaining barrier protective properties, and retaining, or even enhancing, antimicrobial function is encouraging.

Overall, this study demonstrated that keratinocyte exposure to HBD2 did not directly inhibit protease (V8) activity in protecting barrier integrity, but instead modulated cell function and proteomic profiles, associated with enhanced expression levels of ECM proteins, including LAMB1. These proteomic datasets represent a resource that signpost target proteins and pathways for further mechanistic dissection of the key protective processes involved, with a view to the possible future development of novel interventions for AD. Importantly, for therapeutic consideration, concentrations of HBD2 which are sufficient to provide protection against bacterial protease had minimal effects on undamaged monolayers, while protecting protease challenged cells, and peptides could be modified to a simpler linear form while retaining protective function, raising the potential for development of simpler, shorter, functionally-equivalent analogues.

## Materials and methods

### Peptides used

Peptides used for cell stimulation were one of the following: custom synthetic HBD2 (GIGDPVTCLKSGAICHPVFCPRRYKQIGTCGLPGTKCCKKP), scrambled HBD2 (IGKILKHVGLSGYCKGDCTRGPCGPFVITCCQCRKPPPAKT) or linearised HBD2 (GIGDPVTSLKSGAISHPVFSPRRYKQIGTSGLPGTKSSKKP). All peptides provided by Almac, East Lothian, UK.

### Cell maintenance

The immortalised human keratinocyte cell line, HaCaT (CLS GmbH), was maintained in DMEM/F12 media (Life Technologies, USA) with 10% v/v FCS (BioSera, France), 1% penicillin 100 IU/ml (Life Technologies, USA) and streptomycin (Life Technologies, USA) 10 µg/ml with 2 mM L-glutamine (Life Technologies, USA), at 37 °C in an incubator at 95% relative humidity and 5% CO2.

### Cell stimulation

For cell stimulation, HaCaT cells were seeded at 3.5 × 10^6 cells per ml per well in 12-well cell culture flat bottom plates (Costar Corning, USA). These were incubated until monolayer formation at 24 h. Media was removed and cells were washed twice with 1 × Dulbecco’s phosphate-buffered saline (PBS) (Life Technologies, USA) before fresh serum-free media was applied. Additional treatments (as stated for each experiment) were then applied for 24–48 h.

### Damage assays

For damage assays, recombinant V8 (Endoproteinase Glu-C) protease (Worthington biochemicals, USA) was added at the defined concentration for an additional 24 h, following required cell stimulation. Protease damage was assessed from microscopy images acquired using an EVOS fl 2 digital inverted microscope (AMG, USA). Representative images (5 per condition) were analysed, quantifying holes in the monolayer. Damage is expressed as a percentage loss of monolayer integrity, as previously detailed^15^.

### Scratch assays

For scratch assays, HaCaT cells were seeded into 12-well plates (Costar Corning, USA) and stimulated as defined. Cells were then washed with PBS (Life Technologies, USA), and scratches were created by drawing a sterile p200 pipette tip (Gilson, USA) across the monolayer, to produce a linear ‘wound’. Cells were washed with PBS again, before fresh serum-free media (Life Technologies, USA) was added (with additional treatments stated for each experiment). Cells were washed again at 24 h post-scratch. Healing was assessed from images acquired using an EVOS fl 2 digital inverted microscope (AMG, USA). 60 images at 10X magnification were taken per well and tiled together to form each image for analysis. Initial measurements of scratch areas were then used to calculate % healing of scratches from subsequent images, as previously established^40,41^. Images were taken for quantification at 0-, 8- , 24-, 32- and 48-h post-scratch.

### Analysis of fluorescently labelled HBD2

For quantification of TAMRA-HBD2, cells were seeded in to 12-well plates (Costar Corning, USA) and stimulated with TAMRA-HBD2 (Almac, UK) and V8 (Worthington Biochemicals, USA). 5 images were taken per condition, per time point with the EVOS fl 2 digital inverted microscope (AMG, USA). TAMRA quantification was done using Fiji batch macro processing. Script: run("Set Scale…","distance = 0 known = 0 pixel = 1 unit = pixel"); run("32-bit"); run("Subtract Background…", "rolling = 50"); setAutoThreshold("Default dark"); run("Threshold…"); setThreshold(5.0000,1,000,000,000,000,000,000,000,000,000,000.0000); setOption("BlackBackground", false); run("Convert to Mask"); run("Despeckle"); run("Analyze Particles…", "summarize").

### Confocal imaging and 3D rendering

For confocal imaging and 3D rendering, HaCaT cells were seeded into ibidiTREAT 8-well chamber slides (Thistle Scientific, UK) at 3.5 × 10^6 cells/ml. HBD2 (Almac, UK) and V8 (Worthington Biochemicals, USA) were added as detailed. Cells were fixed in 4% paraformaldehyde (PFA) (Sigma-Aldrich, USA) for 10 min at room temperature. Cells were washed twice with PBS (Life Technologies, USA) and permeabilised with 0.1% Triton X-100 (Sigma-Aldrich, USA) for 5 min at room temperature. Methanolic phalloidin conjugated with Alexa488 (Life Technologies, USA) was diluted in 1% Bovine Serum Albumin (Sigma-Aldrich, USA) in PBS and incubated for 30 min at room temperature. Following PBS washed, 1 µg/ml DAPI stain (Thermofisher, USA) in PBS was added and incubated for 5 min at room temperature. Cells were washed a final two times with PBS before imaging. Cells were imaged with the Leica Sp8 confocal microscope at 20 × magnification. Z-stacks were taken at 0.7 μm intervals, with 10 images per stack. 5 fields of view were taken per condition. 3D rendering was then carried out using LASX software. For accessible presentation, red TAMRA fluorescence was artificially recoloured to magenta. Green fluorescence was artificially recoloured to yellow. DAPI was artificially recoloured to cyan.

### Scanning electron microscopy

For scanning electron microscopy, cells were seeded into 35 mm corning coated tissue culture dishes (Costar Corning, USA). Cells were then treated as defined. Following this, cells were fixed with 4%, then 3% glutaraldehyde buffer (Sigma-Aldrich, USA) overnight at 4 °C. Cells were then washed and 3% glutaraldehyde buffer was added for a further overnight incubation at 4 °C. Cells were then further fixed in a solution of 3% glutaraldehyde in 0.1 M sodium cacodylate buffer (pH 7.3) (Sigma-Aldrich, USA) for 2 h. Following washing (consisting of 3 × 10-min incubations in 0.1 M sodium cacodylate buffer), samples were postfixed in 1% osmium tetroxide (TAAB, UK) in 0.1 M sodium cacodylate buffer for 45 min. Cells were washed again before dehydration in graded concentrations of acetone (50, 70, 90 and 100% respectively) (TAAB, UK) was carried out, followed by graded critical point drying using liquid carbon dioxide with a Critical Point Dryer (Polaron, UK). After mounting on aluminium stubs with carbon tabs attached, the specimens were sputter coated with 20 nm gold palladium (TAAB, UK). Images were taken using a Hitachi S-4700 scanning electron microscope (Hitachi, Japan).

### Assessing proteolytic activity

Proteolytic activity of V8 was assessed using fluorescein isothiocyanate (FITC)-labelled casein (Thermofisher, USA) as a substrate. Samples of V8 (Worthington Biochemicals, USA) with various inhibitory candidates were mixed with equal v/v ratio of substrate in a fast optical 96-well reaction plate (Applied Biosystems, USA). Buffer was 25 mM Tris (Thermofisher, USA) with 150 mM NaCl (Thermofisher, USA) (pH 7.2). Fluorescence was recorded at excitation and emission wavelengths at 488 and 530 nm every 5 min for 1 h using a synergy HT plate reader (BioTek, USA). As there was a degree of background fluorescence at the 0-h time point, this measurement was used for normalisation of each respective experiment.

### LC–MS mass spectrometry

LC–MS was performed on a Synapt G2 instrument (Waters Corp., Manchester, UK) with an Acquity UPLC equipped with a reverse phase C4 Aeris Widepore 50 × 2.1 mm HPLC column (Phenomenex, CA, USA) and a gradient of 5–95%B (Mobile phases: A = water + 0.1% formic acid, B = acetonitrile + 0.1% formic acid) over 10 min was employed, as previously established^42^. For LC–MS, samples were typically analysed at 5 μM, and data analysis was performed using MassLynx v4.1 and MaxEnt deconvolution.

### Proteomics and secretomics

For proteomics and secretomics, cells were seeded into 12-well tissue culture coated plates (Costar Corning, USA) and stimulated as stated. 5 separate biological replicates were carried out for each condition, with each replicate seeded from different cell lines stocks with different passage numbers. These replicates were kept separate throughout. For secretomics, media was collected, centrifuged and stored at − 80 °C. Following media collection, the cells were harvested by cell scraping for proteomics analysis. Washed cell pellets were then probe sonicated at 5 μm for 10 s in 40 µl 6 M guanidine hydrochloride (Sigma-Aldrich, USA), 200 mM tris (Sigma-Aldrich, USA). Media was adjusted to 3 M guanidine hydrochloride (Sigma-Aldrich, USA), 100 mM Tris (Sigma-Aldrich, USA), 5 and 10 mM TCEP (Sigma-Aldrich, USA) and CAA (Sigma-Aldrich, USA) respectively. Samples were heated to 95 °C for 5 min. Samples were digested using 0.5 µg lysine C (Wako, Japan) overnight at 37 °C. Samples were then diluted to 1 M guanidine hydrochloride (Sigma-Aldrich, USA) and samples were further digested with 0.5 µg trypsin (Thermofisher, USA) for 4 h at 37 °C. Resulting peptides were acidified by addition of 10% TFA (Sigma-Aldrich, USA). Samples were eluted with 80% acetonitrile (Fisher Scientific, USA), 0.1% TFA (Sigma-Aldrich, USA) and then vacuum-centrifuged. Peptides were separated on an Ultimate 3000 Nano using a C18 packed emitter (IonOptiks, Australia), with a gradient from 4% acetonitrile to 25%. 0.5% acetic acid (Fisher scientific, USA) was present throughout. Peptides were then analysed on a Q Exactive Plus (Thermofisher, USA) in data-dependent mode with MS1 resolution, 70 k scanning 350–1400, and MS2 17.5 K with loop count 24 and NCE26.

Proteomics and secretomics data were processed with MaxQuant version 1.6.3.4 and Uniprot human reference and proteome release 2019_01, with subsequent analyses using Limma Pipeline Proteomics and Metaboanalyst^43,44^. The mass spectrometry proteomics and secretomics data have been deposited to the ProteomeXchange Consortium via the PRIDE ^45^ partner repository with the dataset identifier PXD037844.

### Statistical analysis

All experiments were repeated 3–5 times, unless stated otherwise. Figures show mean values + /- standard error of the mean. Statistical analysis carried out using the GraphPad PRISM9 statistical package. Differences between means of treated and non-treated control groups in different assays were determined by student’s t-test, one-way ANOVA with Dunnett’s multiple comparison post-test, or two-way ANOVA with Bonferroni post-test, with pairing as appropriate. The test used is stated in the respective figure legends. P-values below 0.05 were considered significant throughout. Significant results are marked by asterisks or highlighted in tables. Asterisks are coded by: *p < 0.05, **p < 0.01, ***p < 0.001, ****p < 0.0001.

## Supplementary Information

Supplementary Information.

## Data Availability

Full proteomics and secretomics datasets have been deposited to the ProteomeXchange Consortium (http://proteomecentral.proteomexchange.org) via the PRIDE partner repository with the dataset identifier: PXD037844.
