# Supplementary material for: A mechanistic evaluation of human beta defensin 2 mediated protection of human skin barrier in vitro
Source: Sci Rep. 2023 Feb 8;13:2271. doi: 10.1038/s41598-023-29558-0 (PMC9908873; doi:10.1038/s41598-023-29558-0)
Supplement: Supplementary file 1 — Supplementary Information. [file 41598_2023_29558_MOESM1_ESM.pdf]

Human Beta Defensin-2-mediated protection of human skin barrier *in vitro*; a mechanistic evaluation.

[1]Jennifer R. Shelley*, [1]Brian J. McHugh, [2]Jimi Wills, [1]Julia R. Dorin, [1]Richard Weller, [3]David J. Clarke, [1]Donald J. Davidson

[1] University of Edinburgh Centre for Inflammation Research, Queen's Medical Research Institute, BioQuarter, 47 Little France Crescent, University of Edinburgh, EH16 4TJ, Scotland, UK.

[2] Cancer Research UK Edinburgh Centre, Institute of Genetics and Cancer, The University of Edinburgh, Western General Hospital Campus, Crewe Road, Edinburgh, EH4 2XU, Scotland, UK.

[3] The EastChem School of Chemistry, University of Edinburgh, Joseph Black Building, Brewster Road, Edinburgh, EH9 3FJ, UK.

* Corresponding author

Corresponding author – current contact details

Dr Jennifer R. Shelley

Address: The Commonwealth Building, The Hammersmith Hospital, Du Cane Road, London, W12 0NN

E-mail: jennifer.shelley@imperial.ac.uk

Phone: 02075895111

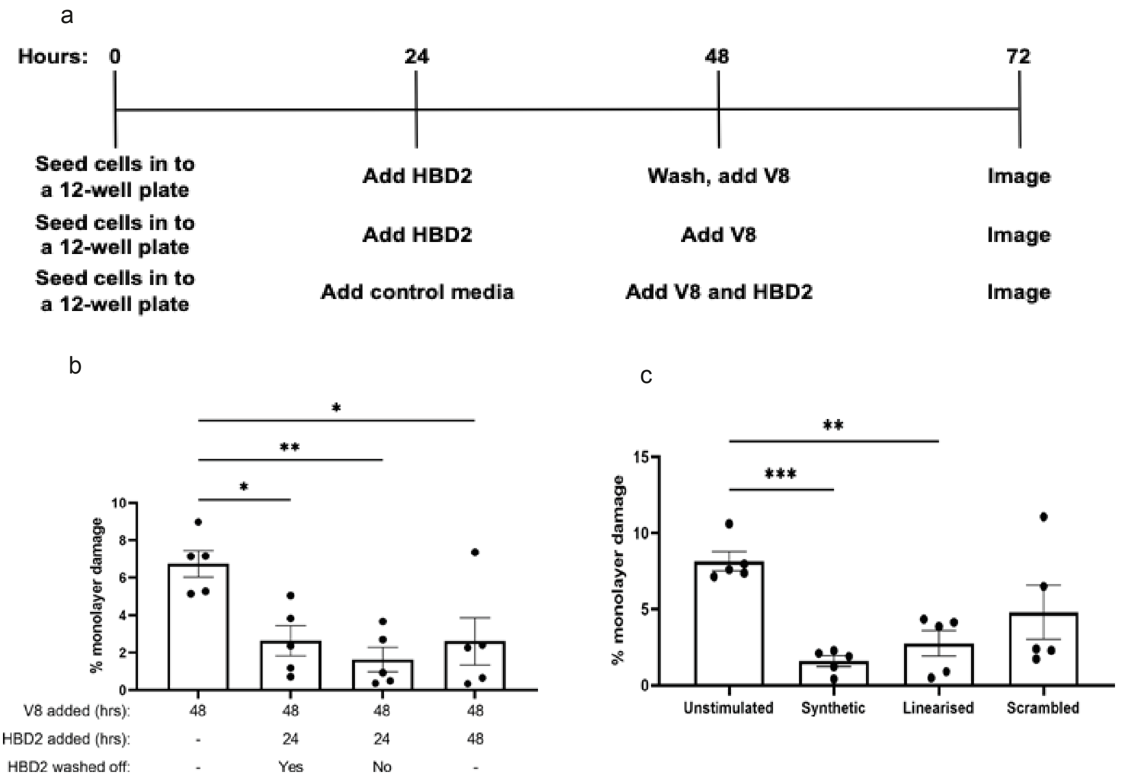

Supplementary figure S1. HBD2-mediated protection against V8 is maintained irrespective of peptide application timeline and folding

(a) Alternative timelines of damage assays, with treatment conditions indicated

(b) HaCaT cells were treated with 0.5 µg/ml synthetic HBD2 or vehicle control. HBD2 was added at 24 hours after seeding cells and then washed off 24 hours later, or not washed off or not added until 48 hours after seeding cells (concurrently with V8). 5 µg/ml recombinant V8 was added for 24 hours before quantification of % monolayer damage from 5 random fields of view per condition. Data shown as mean ± SEM for n = 5. Statistical analysis by paired one-way ANOVA with RM multiple comparisons post-test. * $P < 0.05$, ** $P < 0.01$

(c) HaCaT cells were treated with 0.5 µg/ml synthetic, linearised or scrambled HBD2 or vehicle control (unstimulated) for 24 hours, followed by exposure to recombinant 5 µg/ml V8 for a further 24 hours, without washing. 5 random fields of view were imaged per condition for quantification of % monolayer damage. Data shown as mean ± SEM for n = 5. Statistical analysis by paired one-way ANOVA with RM multiple comparisons post-test. ** $P < 0.01$ *** $P < 0.005$

Peptide	Sequence	Theoretical average neutral mass (reduced) / Da	Theoretical average neutral mass (oxidised) / Da	Observed average neutral mass according to LC-MS / Da
Synthetic	GIGDPVT**C**LKSGAI**C**HPVF**C**PR RYKQIGT**C**GLPGTK**CC**KKP	4334.2	4328.2	4328.8
Scrambled	IGKILKHVGLSGY**C**KGD**C**TRGP **C**GPFVIT**CC**Q**C**RKPPPAKT	4334.2	4328.2	4332.22
Linearised	GIGDPVT*S*LKSGAI*S*HPVF*S*PRR YKQIGT*S*GLPGTK*SS*KKP	4237.82	4237.82	4237.7

Supplementary table S1. Theoretical masses of HBD2 peptides used

Theoretical masses of various HBD2 peptide forms with oxidised or reduced cysteines, alongside the calculated observed masses from LC-MS analysis

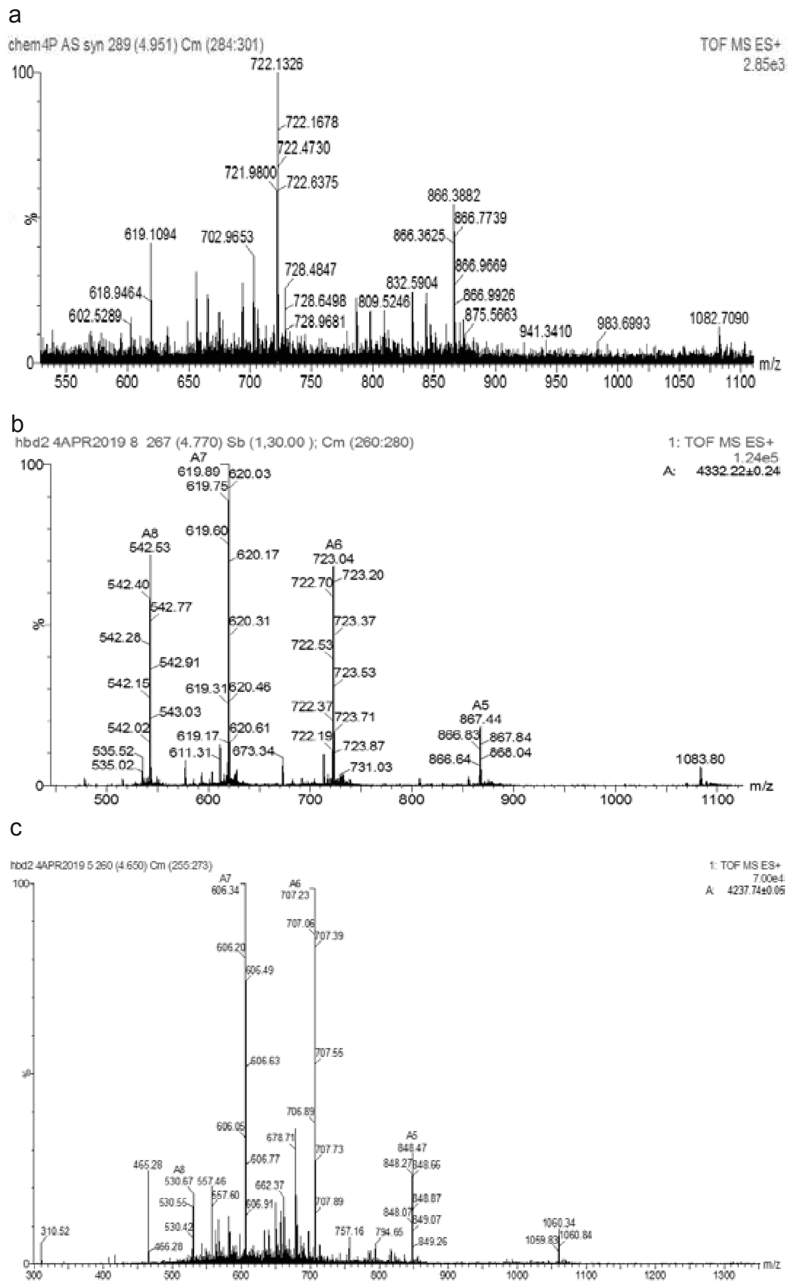

Supplementary figure S2. LC-MS analysis of synthetic, scrambled and linearised HBD2

(a-c) Accurate mass measurements of (b) synthetic, (c) scrambled and (d) linearised HBD2, performed by LC-MS. All peptides displayed a molecular mass consistent with the theoretical mass, given the known amino acid sequence. Synthetic HBD2 displayed a molecular mass consistent with the presence of 3 disulfide bonds. Scrambled HBD2 displayed a average mass consistent with a mix of oxidised forms of the peptide.

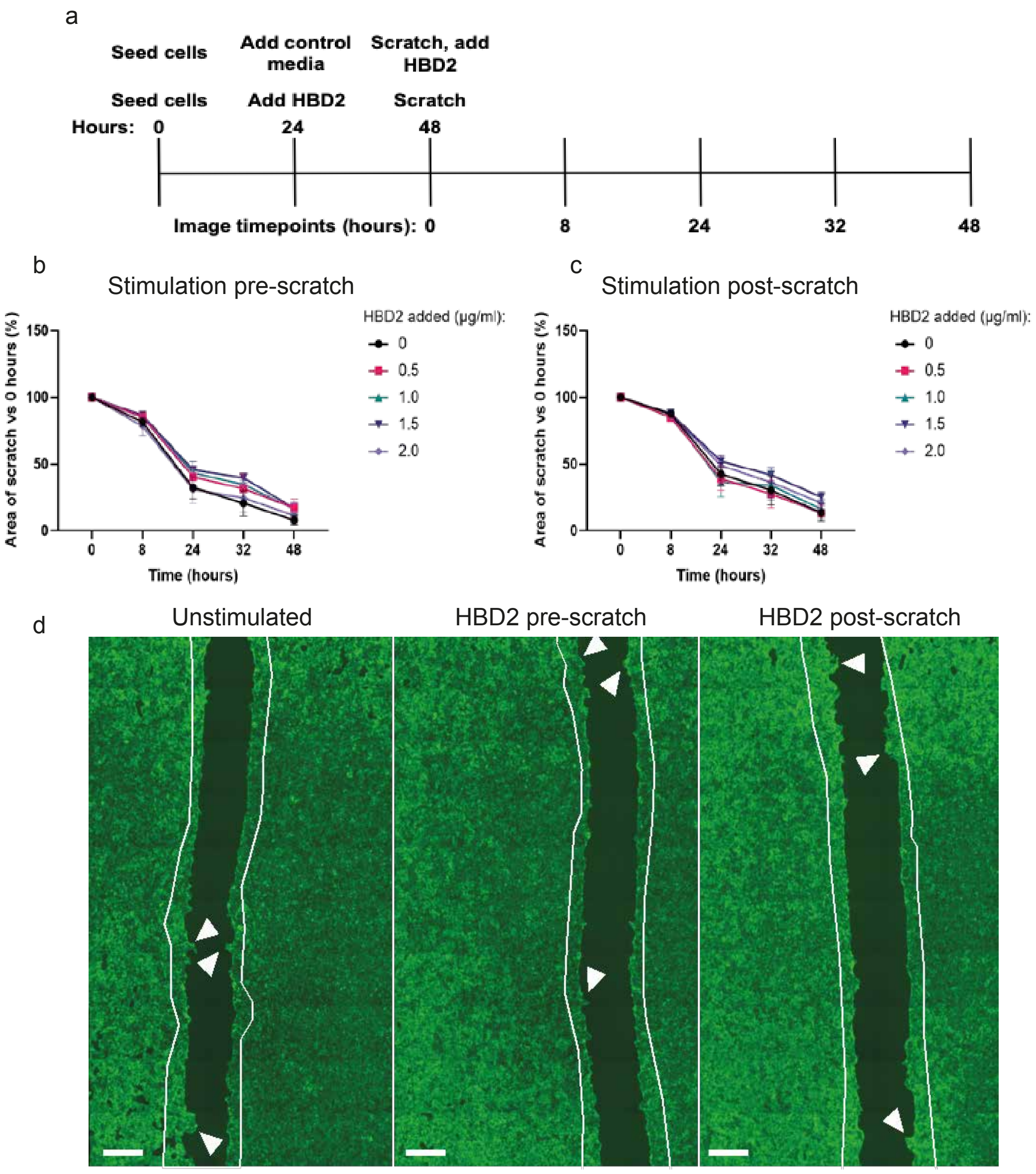

Supplementary figure S3. HBD2 does not modulate HaCaT cell scratch wound healing

(a) Timeline for scratch assays, indicating pre- and post-scratch stimulation and imaging timepoints.

(b-d) HaCaT cells seeded into 12-well plates were grown until confluency. 0.5 µg/ml HBD2 was added at either 24 (stimulation pre-scratch) or 48-hours (stimulation post-scratch) after seeding. A p100 pipette tip was drawn vertically down the cell monolayer to introduce a 'wound' or 'scratch' at 48 hours post-seeding. The monolayer was immediately imaged (0-hour timepoint), with subsequent images taken at 8-, 24-, 32- and 48-hours post-scratch. Cells were incubated in serum-free media. (b+c) Data show the area of the scratch at each timepoint

as a percentage of the scratch area at the 0-hour timepoint. Each point represents mean ± SEM for n = 5. (d) Representative images of scratch healing after 24 hours. Alexa488 Phalloidin stained fixed cell samples. Scale bar = 400 µm. 60 images at 10x magnification taken per well, then stitched together. White lines indicate scratch border at 0-hours. Arrowheads indicate 'leading edge' migration of the cell monolayer.

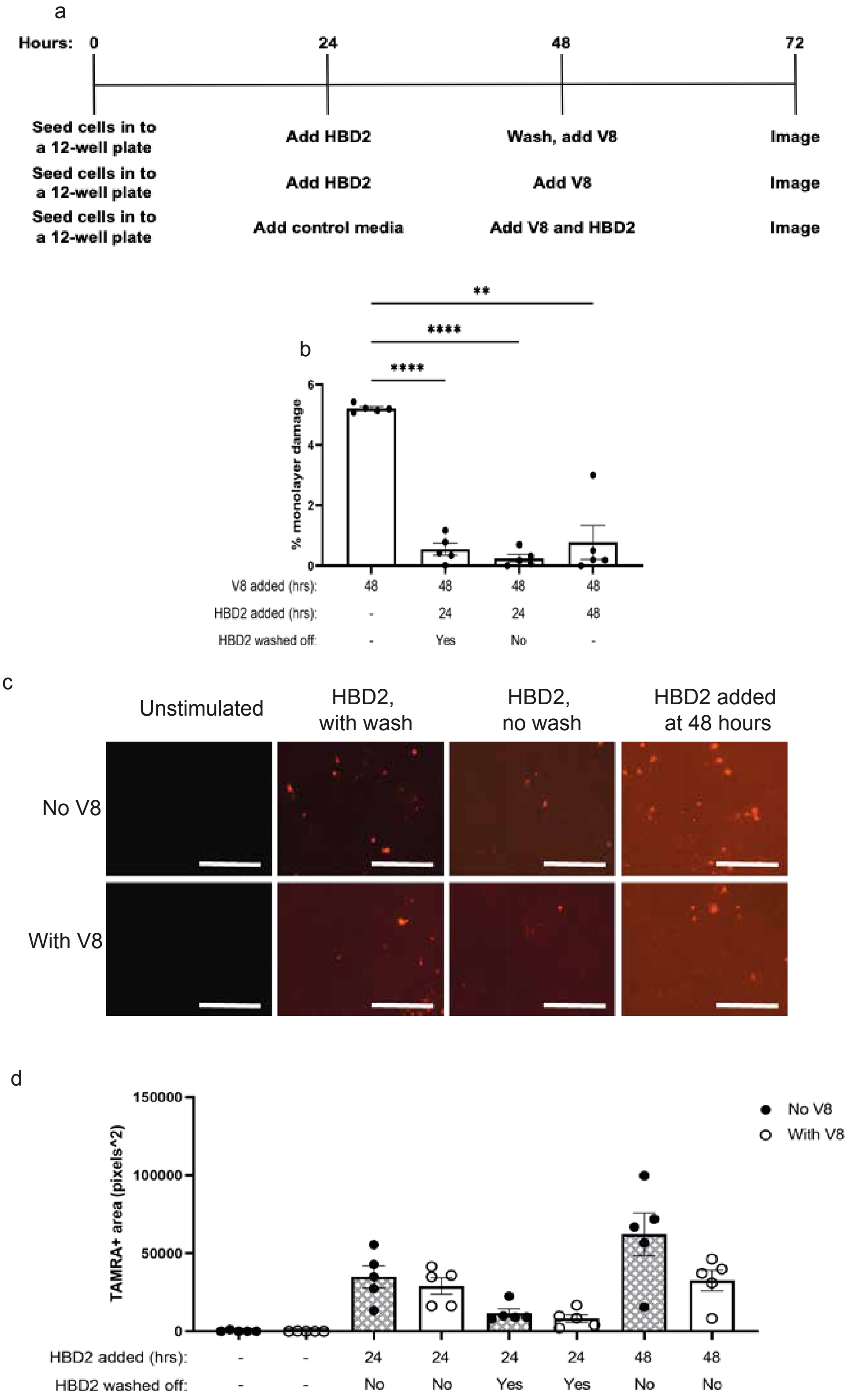

Supplementary figure S4. Visualisation of fluorescently labelled HBD2

(a) Timelines of damage assays, with treatment conditions indicated

(b-d) HaCaT cells were treated with 0.5 µg/ml synthetic TAMRA-labelled HBD2 or vehicle control. Peptide was added 24 hours after seeding cells and then washed off 24 hours later, or not washed off or not added until 48 hours after seeding cells (concurrently with V8). 5 µg/ml recombinant V8 was added for 24 hours before quantification of % monolayer damage from 5 random fields of view per condition per time point were imaged. (b) Data shown as mean ± SEM for n = 5. Statistical analysis by paired one-way ANOVA with RM multiple comparisons post-test. ** $P < 0.01$ **** $P < 0.0001$. (c) Representative images of TAMRA fluorescence for each condition. Scale bar represents 100 µm. (d) Total area of fluorescence (in pixels) was batch quantified using a Fiji macro (detailed in methods) for each field of view, at each time point in each condition. Average fluorescent area for the 5 fields of view in each replicate represented. Data shown as mean ± SEM for n=5. Statistical analysis by paired one-way ANOVA with RM multiple comparisons post-test.

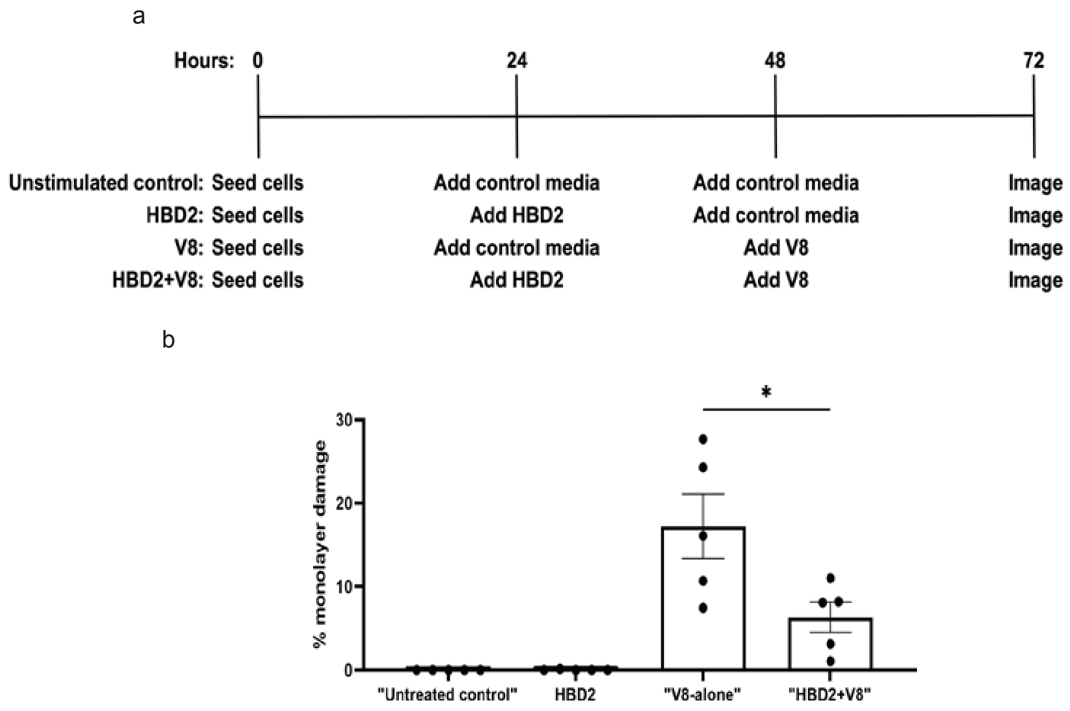

Supplementary figure S5. Cells used for secretomics, and proteomics demonstrated the protective phenotype of HBD2

(a) Timelines of damage assays, with treatment conditions indicated (b) HaCaT cell monolayers were seeded in 12-well plates, then treated, according to the associated timeline, with 0.5 µg/ml HBD2 or vehicle control for 24 hours, then exposed to 5 µg/ml V8, or vehicle control, for a further 24 hours, all in serum free media. Cells were imaged and the damage quantified before harvesting of the cell pellet and supernatants for analyses. Quantification of % monolayer damage shown as mean ± SEM for n = 5. Statistical analysis carried out using paired t test. * $P < 0.05$

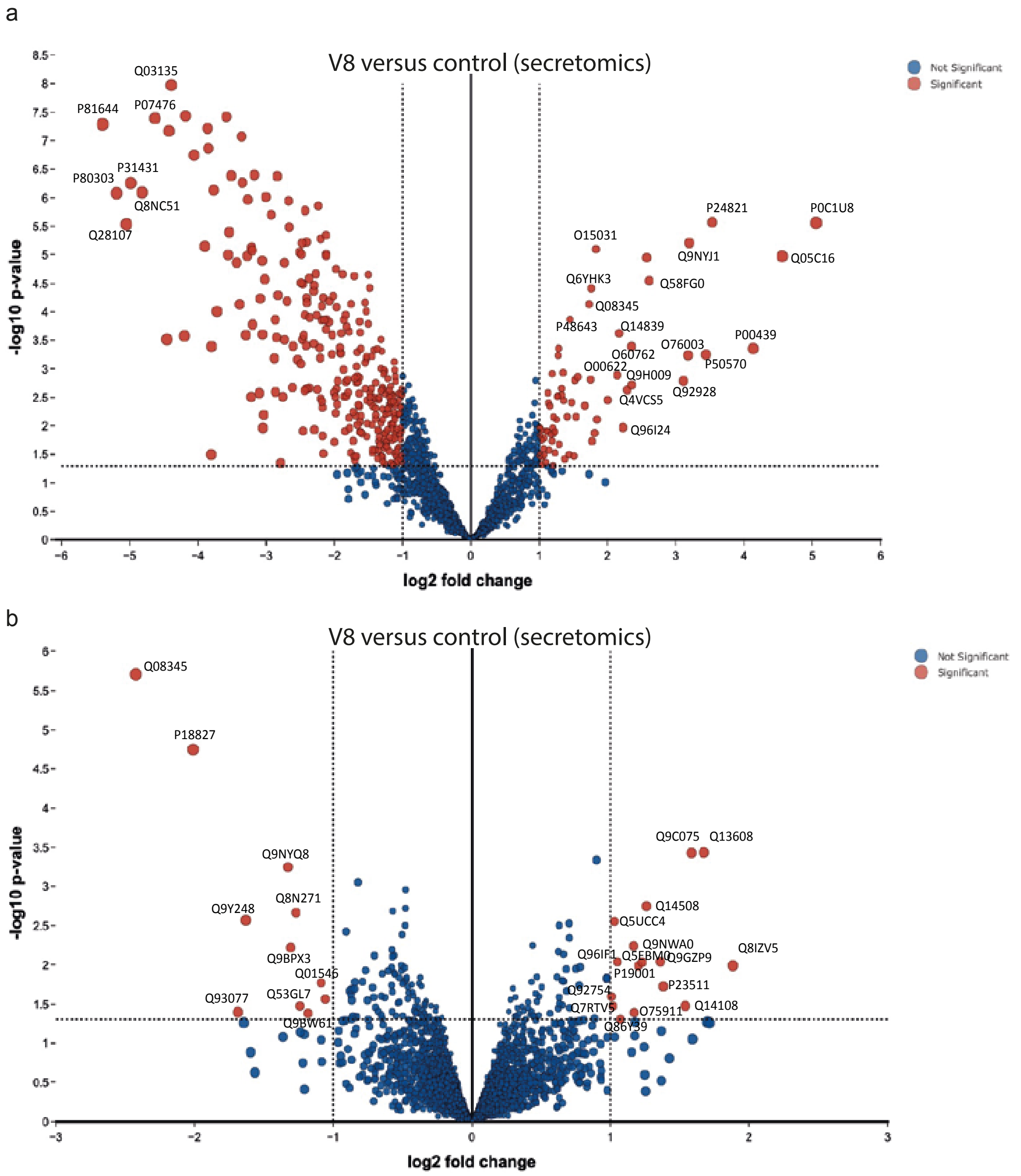

Supplementary figure S6. Secretomic and proteomic analyses of "V8-alone"-damaged HaCaT cells

HaCaT cell monolayers were treated with 5 µg/ml V8 for 24 hours, 48 hours after seeding. Supernatants (a) and cells (b) were then collected, centrifuged at 3000 rpm for 5 minutes, and stored at -80 ºC prior to analysis. Data representative of n=5. Log fold changes and p values generated using LIMMA pathway[37].

(a) Volcano plot generated from secretomics analysis of V8-stimulated HaCaT cells compared to control vehicle-treated cells. Red points indicate significant points, blue indicate nonsignificant points.

(b) Volcano plot generated proteomics analysis of V8-stimulated HaCaT cells compared to control vehicle-treated cells. Red points indicate significant points, blue indicate nonsignificant points.

Protein code	Gene	Log2 fold change	p.mod	Protein name
P0C1U8	SSPA	5.05	0.00	Glutamyl endopeptidase
Q05C16	LRRC63	4.56	0.00	Leucine-rich repeat-containing protein 63
P00439	PAH	4.13	0.00	Phenylalanine-4-hydroxylase
P24821	TNC	3.53	0.00	Tenascin
P50570	DNM2	3.44	0.00	Dynamin-2
Q9NYJ1	COA4	3.20	0.00	Cytochrome c oxidase assembly factor 4 homolog, mitochondrial
O76003	GLRX3	3.18	0.00	Glutaredoxin-3
Q9H0U4	RAB1B	3.11	0.00	Putative Ras-related protein Rab-1C
Q58FG0	HSP90AA5P	2.61	0.00	Putative heat shock protein HSP 90-alpha A5
Q9H009	NACA2	2.35	0.00	Nascent polypeptide-associated complex subunit alpha-2
O60762	DPM1	2.35	0.00	Dolichol-phosphate mannosyltransferase subunit 1
Q4VCS5	AMOT	2.29	0.00	Angiomotin
Q96I24	FUBP3	2.23	0.01	Far upstream element-binding protein 3
Q14839	CHD4;CHD5	2.17	0.00	Chromodomain-helicase-DNA-binding protein 4
O00622	CCN1	2.14	0.00	CCN family member 1
O15031	PLXNB2	1.83	0.00	Plexin-B2
Q6YHK3	CD109	1.76	0.00	CD109 antigen
Q08345	DDR1	1.73	0.00	Epithelial discoidin domain-containing receptor 1
P48643	CCT5	1.45	0.00	T-complex protein 1 subunit epsilon
Q9BRK5	SDF4	-2.83	0.00	45 kDa calcium-binding protein

Q8WVN6	SECTM1	-3.17	0.00	Secreted and transmembrane protein 1
B4E1Z4	CFB	-3.30	0.00	Complement factor B
Q32MZ4	LRRFIP1	-3.35	0.00	Leucine-rich repeat flightless-interacting protein 1
Q14847	LASP1	-3.36	0.00	LIM and SH3 domain protein 1
Q86UP2	KTN1	-3.39	0.00	Kinectin
O75635	SERPINB7	-3.43	0.00	Serpin B7
Q7Z406	MYH14	-3.51	0.00	Myosin-14
P04156	PRNP	-3.54	0.00	Major prion protein
P26038	MSN	-3.56	0.00	Moesin
Q15056	EIF4H	-3.58	0.00	Eukaryotic translation initiation factor 4H
P62328	TMSB4X	-3.72	0.00	Thymosin beta-4
O76070	SNCG	-3.77	0.00	Gamma-synuclein
Q86TE4	LUZP2	-3.80	0.00	Leucine zipper protein 2
P02777	Bovine PF4	-3.80	0.03	Bovine Platelet factor 4
Q02818	NUCB1	-3.85	0.00	Nucleobindin-1
P51858	HDGF	-3.86	0.00	Hepatoma-derived growth factor
O75821	EIF3G	-3.90	0.00	Eukaryotic translation initiation factor 3 subunit G
P51572	BCAP31	-4.06	0.00	B-cell receptor-associated protein 31
Q00688	FKBP3	-4.18	0.00	Peptidyl-prolyl cis-trans isomerase FKBP3
Q9HB71	CACYBP	-4.20	0.00	Calcyclin-binding protein
Q03135	CAV1	-4.39	0.00	Caveolin-1
P35241	RDX	-4.42	0.00	Radixin
P06753	TPM3	-4.45	0.00	Tropomyosin alpha-3 chain
P07476	IVL	-4.63	0.00	Involucrin
Q8NC51	SERBP1	-4.82	0.00	Plasminogen activator inhibitor 1 RNA-binding protein

P31431	SDC4	-4.98	0.00	Syndecan-4
Q28107	Bovine F5	-5.04	0.00	Coagulation factor V
P80303	NUCB2	-5.19	0.00	Nucleobindin-2
P81644	Bovine APOA2	-5.40	0.00	Bovine Apolipoprotein A-II

Supplementary table S2. Secretomic analysis from "V8-only" conditions

HaCaT cell monolayers were treated with 5 µg/ml V8 for 24 hours, 48 hours after seeding. Supernatants were then collected, centrifuged at 3000 rpm for 5 minutes and stored at -80 ºC prior to analysis. n=5 per condition. Log fold changes and p values generated using LIMMA pathway[37]. Table shows proteins with the greatest differences in detection levels between control and V8 treated conditions, largest to smallest, with Uniprot protein code, gene code, fold change, p.mod value, protein name and brief description of function. Protein descriptions made using UniProt database information[39].

Protein code	Gene	Log2 fold change	p.mod	Protein name
Q8IZV5	RDH10	1.88	0.01	Retinol dehydrogenase 10
Q13608	PEX6	1.67	0.00	Peroxisome assembly factor 2
Q9C075	KRT23	1.58	0.00	Keratin, type I cytoskeletal 23
Q14108	SCARB2	1.54	0.03	Lysosome membrane protein 2
P23511	NFYA	1.38	0.02	Nuclear transcription factor Y subunit alpha
Q14508	WFDC2	1.26	0.00	WAP four-disulfide core domain protein 2
Q9GZP9	DERL2	1.36	0.01	Derlin-2
Q5EBM0	CMPK2	1.23	0.01	UMP-CMP kinase 2, mitochondrial
P19001	Murine KRT19	1.20	0.01	Murine Keratin, type I cytoskeletal 19
O75911	DHRS3	1.17	0.04	Short-chain dehydrogenase/reductase 3
Q9NWA0	MED9	1.17	0.01	Mediator of RNA polymerase II transcription subunit 9
Q86Y39	NDUFA11	1.07	0.05	NADH dehydrogenase [ubiquinone] 1 alpha subcomplex subunit 11
Q96IF1	AJUBA	1.05	0.01	LIM domain-containing protein ajuba
Q5UCC4	EMC10	1.03	0.00	ER membrane protein complex subunit 10
Q7RTV5	AAED1	1.02	0.03	Peroxiredoxin-like 2C
Q92754	TFAP2C	1.01	0.03	Transcription factor AP-2 gamma
Q93077	HIST1H2AC	-1.69	0.04	Histone H2A type 1-C
P18827	SDC1	-2.01	0.00	Syndecan-1
Q08345	DDR1	-2.42	0.00	Epithelial discoidin domain-containing receptor 1
Q9Y248	GINS2	-1.63	0.00	DNA replication complex GINS protein PSF2
Q8N271	PROM2	-1.27	0.00	Prominin-2
Q9BPX3	NCAPG	-1.31	0.01	Condensin complex subunit 3

Q9NYQ8	FAT2	-1.33	0.00	Protocadherin Fat 2
Q53GL7	PARP10	-1.24	0.03	Protein mono-ADP-ribosyltransferase PARP10
Q01546	KRT76	-1.09	0.02	Keratin, type II cytoskeletal 2 oral
Q9BW61	DDA1	-1.18	0.04	DET1- and DDB1-associated protein 1
Q6YHK3	CD109	-1.06	0.03	CD109 antigen

Supplementary table S3. Proteomic analysis of V8-damaged cells

HaCaT cell monolayers were treated with 5 µg/ml V8 for 24 hours, 48 hours after seeding. The cells were then washed rigorously with PBS and scraped from the well surface. The cell pellet was washed an additional three times with PBS in suspension. Dry cell pellet stored at -80 ºC prior to analysis. n=5 per condition. Log fold changes and p values generated using LIMMA pathway[37]. Table shows proteins with the greatest differences in detection levels between control and V8 treated conditions, largest to smallest, with Uniprot protein code, gene code, fold change, p.mod value, protein name and brief description of function. Protein descriptions made using UniProt database information[39].
